# High-Carbohydrate Diet Consumption Poses a More Severe Liver Cholesterol Deposition than a High-Fat and High-Calorie Diet in Mice

**DOI:** 10.3390/ijms241914700

**Published:** 2023-09-28

**Authors:** Linyu Zhang, Xin Li, Xiangyan Liu, Xiaoran Wu, Qiurong Xu, Jianyu Qu, Xiaowen Li, Yuanyuan Zhu, Lixin Wen, Ji Wang

**Affiliations:** 1 Hunan Engineering Research Center of Livestock and Poultry Health Care, College of Veterinary Medicine, Hunan Agricultural University, Changsha 410128, China; zhanglinyu@stu.hunau.edu.cn (L.Z.); lixin0822@stu.hunau.edu.cn (X.L.); liuxiangyan@stu.hunau.edu.cn (X.L.); xrwu0821@163.com (X.W.); x17393118112@163.com (Q.X.); qjy@stu.hunau.edu.cn (J.Q.); lxw0630@stu.hunau.edu.cn (X.L.); hong6002@163.com (Y.Z.); 2 Animal Nutritional Genome and Germplasm Innovation Research Center, College of Animal Science and Technology, Hunan Agricultural University, Changsha 410128, China

**Keywords:** high-fat diet, high carbohydrate diet, high caloric diet, intestinal microbiota, non-alcoholic fatty liver disease

## Abstract

In the past few decades, many researchers believed that a high-fat and high-calorie diet is the most critical factor leading to metabolic diseases. However, increasing evidence shows a high-carbohydrate and low-fat diet may also be a significant risk factor. It needs a comprehensive evaluation to prove which viewpoint is more persuasive. We systematically compared the effects of high-fat and high-calorie diets and high-carbohydrate and low-fat ones on glycolipid metabolism in mice to evaluate and compare the effects of different dietary patterns on metabolic changes in mice. Sixty 8-week-old male C57BL/6 mice were divided into four groups after acclimatization and 15% (F-15), 25% (F-25), 35% (F-35), and 45% (F-45) of their dietary energy was derived from fat for 24 weeks. The body weight, body-fat percentage, fasting blood glucose, lipid content in the serum, and triglyceride content in the livers of mice showed a significantly positive correlation with dietary oil supplementation. Interestingly, the total cholesterol content in the livers of mice in the F-15 group was significantly higher than that in other groups (*p* < 0.05). Compared with the F-45 group, the mRNA expression of sterol synthesis and absorption-related genes (e.g., *Asgr1*, *mTorc1*, *Ucp20*, *Srebp2*, *Hmgcr*, and *Ldlr*), liver fibrosis-related genes (e.g., *Col4a1* and *Adamts1*) and inflammation-related genes (e.g., *Il-1β* and *Il-6*) were significantly higher in the F-15 group. Compared with the F-45 group, the relative abundance of *unclassified_f_Lachnospiraceae* and *Akkermansia* was decreased in the F-15 group. While *unclassified_f_Lachnospiraceae* and *Akkermansia* are potentially beneficial bacteria, they have the ability to produce short-chain fatty acids and modulate cholesterol metabolism. In addition, the relative abundance of *unclassified_f_Lachnospiraceae* and *Akkermansia* was significantly positively correlated with fatty acid transporters expression and negatively correlated with that of cholesteryl acyltransferase 1 and cholesterol synthesis-related genes. In conclusion, our study delineated how a high-fat and high-calorie diet (fat supplied higher than or equal to 35%) induced obesity and hepatic lipid deposition in mice. Although the high-carbohydrate and low-fat diet did not cause weight gain in mice, it induced cholesterol deposition in the liver. The mechanism is mainly through the induction of endogenous synthesis of cholesterol in mice liver through the ASGR1-mTORC1-USP20-HMGCR signaling pathway. The appropriate oil and carbon water ratio (dietary energy supply from fat of 25%) showed the best gluco-lipid metabolic homeostasis in mice.

## 1. Introduction

The consumption of a high-fat and high-calorie diet shifts the energy balance towards ectopic lipid accumulation, especially in the liver, resulting in inflammation, oxidative stress, and fibrosis, causing organ damage and loss of function [1]. Since the 20th century, a high-fat and high-calorie diet has been established as one of the key causes of obesity and glucose and lipid metabolism disorders [2]. The high-fat and high-calorie diet is a model to investigate lipid-induced insulin resistance, and evidence shows that such short-term diets reduce insulin sensitivity in healthy young men [3]. In addition, these diets minimize the rate of synthesis and turnover of primary bile salts in humans, affecting cholesterol clearance from the body [4]. High-fat and high-calorie diets have been used in animal experiments to construct disease models, such as atherosclerosis, type 2 diabetes mellitus (T2DM), and non-alcoholic fatty liver disease (NAFLD) models [5,6,7,8,9].

Despite previous confirmation of high fat being a significant factor in metabolic diseases, in recent years, more and more studies have suggested that excessive high carbohydrate intake equally contributes to metabolic syndrome. A high-carbohydrate and low-fat (HCLF) diet is as harmful as a high-fat and high-calorie diet in the development and progression of liver injury in mice [10,11], and long-term consumption of such a diet induces NAFLD in mice [12]. More importantly, feeding a high-fat, high-carbohydrate diet causes liver fibrosis and nonalcoholic steatohepatitis (NASH) in mice [13]. Tomasello et al. [14] showed that the ‘Western diet’, particularly a low-fiber high-fat/high-carbohydrate diet, could lead to severe dysbiosis. Moreover, Sartorius et al. [15] suggested that the assumption that no carbohydrates are associated with obesity is potentially erroneous. In addition, the HCLF diet increased triglyceride (TG) content and insulin levels in the serum [16,17]. More interestingly, T2DM patients receiving a long-term high-fat and low-carbohydrate diet showed more clinically meaningful improvements in glycemic control and body weight compared to the HCLF diet [18]. However, hardly any study directly compared high-fat, high-calorie, and HCLF diets to elucidate the differential effects of the two diets on glucose and lipid metabolism disorders.

It is well known that dietary components substantially alter intestinal physiology and specifically modulate the diversity of gut microbiota [19]. The intestinal tract is the largest barrier between a person and the environment. The intestinal microbiota is responsible for recovering energy from food, providing hosts with vitamins, and providing a barrier function against exogenous pathogens [20]. There is considerable evidence of a relationship between dietary fats and colonic microbiota composition. The obesity-related ecological imbalance is directly associated with the high-fat diet (HFD) and manifests in a reduced overall microbiota count, a shift in bacteria species abundance, and an overall increase in gut permeability [20,21]. Also, studies have shown that a high-calorie diet in rats induced decreased bowel sounds and fecal microbial imbalance [22]. The increase in unfavorable bacteria (such as *Lachnoclostridium* and *Desulfovibrio*) was strongly associated with a disturbance of glucose and lipid metabolism [23].

In this study, we used mice to set up four common dietary patterns (15–45% of energy from fat) to explore the differences and molecular mechanisms underlying a high-fat and high-calorie diet and an HCLF diet on glucose, gut microbiota, and lipid metabolism in the body.

## 2. Results

### 2.1. Changes in Growth and Serum Glucose Levels in Mice

**Figure 1:**
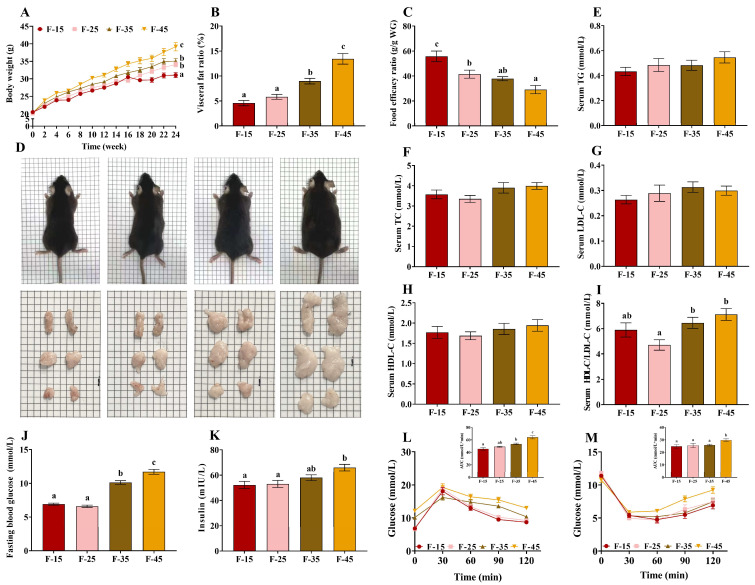
Effects of energy supply from fat on growth performance and glucose levels in mice. (**A**) Body weight curve. (**B**) Visceral fat percentage. (**C**) Food efficiency ratio. (**D**) Representative images of male mice from different experimental groups (upper row) and their subcutaneous fat, perirenal fat, and epididymal fat pads (bottom row) after 24 weeks of respective diets. (**E**) Serum TG content. (**F**) Serum TC content. (**G**) Serum LDL-C content. (**H**) Serum HDL-C content. (**I**) Ratio of HDL-C/LDL-C. (**J**) Final fasting blood glucose levels. (**K**) Serum insulin content. (**L**) Blood glucose levels in the oral glucose tolerance test (OGTT) and the area under the curve (AUC) values. (**M**) Blood glucose levels in the insulin tolerance test (ITT) and the AUC values. Data are expressed as the mean ± SEM. For all groups, n = 15. Different superscript letters (a–c) indicate significant differences.

After 24 weeks, the body weight of mice increased significantly with the increase of the supply of fat, and the weight of mice was highest in the F-45 group (*p* < 0.05) (Figure 1A,D); however, the trend of changes in the feed conversion ratio was contradictory (Figure 1C). Accumulation of visceral fat was significantly higher in the F-35 and F-45 groups than in the F-15 and F-25 groups (*p* < 0.01) (Figure 1B,D). No significant differences were observed in the levels of serum HDL-C, LDL-C, TG, and TC among the four groups (*p* > 0.05); however, the ratio of HDL-C/LDL-C in the serum was significantly lower in the F-25 group than in the F-35 and F-45 groups (*p* < 0.01) (Figure 1E–I). In addition, blood glucose levels were significantly higher in the F-35 and F-45 groups than in the F-15 and F-25 groups (*p* < 0.01), and fasting insulin levels were significantly higher in the F-45 group than in the F-15 and F-25 groups (*p* < 0.05) (Figure 1J,K). There was a significant increase in the oral glucose tolerance test (OGTT) in the F-35 group than in the F-15 group (*p* < 0.05). Moreover, OGTT and ITT were significantly higher in the F-45 group than in the other groups (*p* < 0.05) (Figure 1L,M). These results suggest that dietary energy supplied from fat above 35% increases blood glucose levels and impairs glucose tolerance. 

### 2.2. High-Fat and High-Carbohydrate Diet-Induced Hepatic Steatosis and Impaired Glucose Regulation in Mice

**Figure 2:**
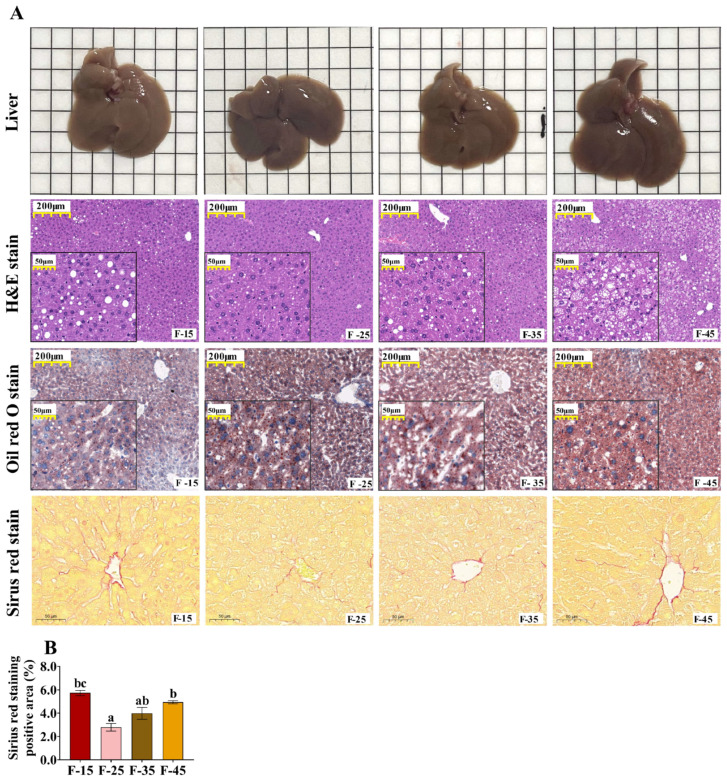
Effects of energy supply from fat on hepatic steatosis and fibrosis in mice. (**A**) Representative images of the liver tissue of male mice from different experimental groups (upper row), H&E-stained (second row), oil red O-stained (third row), and Sirius red staining (bottom row) (magnification 50×, scale bar = 200 μm; magnification 200×, scale bar = 50 μm). (**B**) Positive areas stained with Sirius red were quantitated using digital image analysis of liver sections of mice. n = 3 for each group. Different superscript letters (a–c) indicate significant differences.

**Figure 3:**
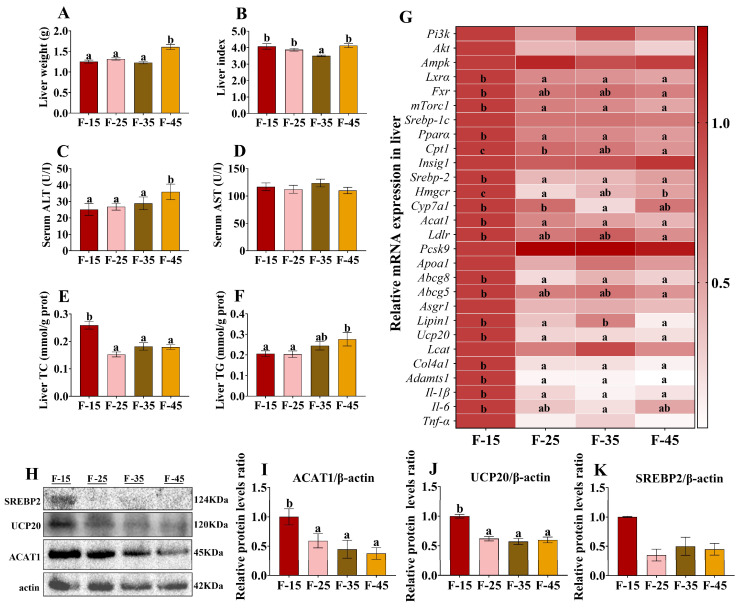
Effects of energy supply from fat on the expression of genes and proteins related to cholesterol synthesis in the livers of mice. (**A**) Liver weight. (**B**) Liver index. (**C**) Serum ALT content. (**D**) Serum AST content. (**E**) Liver TC content. (**F**) Liver TG content. (**G**) The mRNA expression levels of genes associated with lipid synthesis and decomposition in the livers of mice n = 5 for each group. (**H**) Western blot protein bands. (**I**) protein expression of ACAT1. (**J**) protein expression of UCP20. (**K**) protein expression of SREBP2. Data are presented as the mean ± SEM. For all groups, n = 15. Different superscript letters (a–c) indicate significant differences.

H&E and oil red O staining revealed a higher accumulation of lipid droplets in the liver in the F-35 and F-45 groups than in the F-25 groups (Figure 2A). The results of Sirius red staining showed that the level of fibrotic tissue in the livers of mice in group F-25 was significantly lower than that in groups F-15 and F-45, and the level of fibrotic tissue in the livers of mice in group F-35 was significantly lower than that in group F-15 (*p* < 0.05), but there was no difference between groups F-15 and F-45. (*p* > 0.05) (Figure 2A,B). The liver weight, liver index, and liver TG content were significantly higher in the F-45 group than in the F-15 and the F-25 groups (*p* < 0.05) (Figure 3A,B,F). These results indicated the development of hepatic steatosis marked by high hepatic TG levels in mice fed a high-fat and high-calorie diet. Interestingly, the HCLF-diet (F-15) group had increased hepatic deposition of total cholesterol in the liver (*p* < 0.01) (Figure 3E). The activity of AST in the serum was not significantly different among the four groups (Figure 3D). However, the activity of ALT in the serum was significantly higher in the F-45 group than in the F-15 group (*p* < 0.05) (Figure 3C). These results suggest that a high-fat and high-calorie diet adversely affects liver function by increasing serum ALT levels.

Although the HCLF diet did not cause substantial weight gain, it induced more cholesterol deposition in the liver compared with the high-fat and high-calorie diet. To examine the underlying mechanisms, RT-qPCR was performed to detect the expression of mRNAs related to fatty acid metabolism and cholesterol anabolism in the liver and small intestine (Figure 3G). The mRNA levels of *Ucp20*, *mTorc1*, *Lxrα*, *Fxr*, and *Srebp2* (five regulatory factors of lipid metabolism); *Pparα* and *Cpt1* (two lipid-decomposition-associated genes); *Hmgcr* (the rate-limiting enzyme associated with cholesterol synthetic); *Aact1*, *Abcg8*, and *Abcg5* (related genes of cholesterol storage and transport protein); and *Ldlr* were markedly higher in the F-15 group than in the F-45 group (*p* < 0.05). However, no significant differences were found in the mRNA levels of *Pi3k*, *Akt*, *Srebp-1c*, *Ampk*, *Insig1*, *Pcsk9, Apoa1, Asgr1*, and *Lact* (*p* > 0.05). In addition, the expressions of genes associated with fatty acid decomposition and cholesterol storage and transport in the liver were significantly lower in the F-15 group than in the F-25 and F-35 groups (*p* < 0.05). Western blot analysis showed that the protein expression of ACAT1 and UCP20 in the livers of mice in the F-15 group was significantly higher than that in the other three groups (*p* < 0.05) (Figure 3H–J), while the protein expression of SREBP2 increased although it was not significantly different from that in the other three groups (*p* > 0.05) (Figure 3H,K). Protein expression results were consistent with gene results. These results indicate that compared with a high-fat and high-calorie diet, intake of the HCLF diet can significantly up-regulate the expression of cholesterol regulators and rate-limiting enzymes of cholesterol synthesis in the livers of mice, thereby increasing the risk of hepatic cholesterol deposition. We further examined gene expression associated with liver fibrosis and inflammation. As shown in Figure 3G, the mRNA expression levels of *Col4a1*, *Adamts1*, and *Il-1β* in the livers of mice in the F-15 group were significantly higher than those in the remaining three groups (*p* < 0.05), and the mRNA expression level of *Il-6* was significantly higher than that in the F-35 group, whereas there was no significant difference in the mRNA expression levels of *Tnf-α* among the groups (*p* > 0.05), but the expression in the F-15 group was still the highest.

### 2.3. The Structure and Lipid Absorption of the Small Intestine Are Impaired by a High-Fat Diet

**Figure 4:**
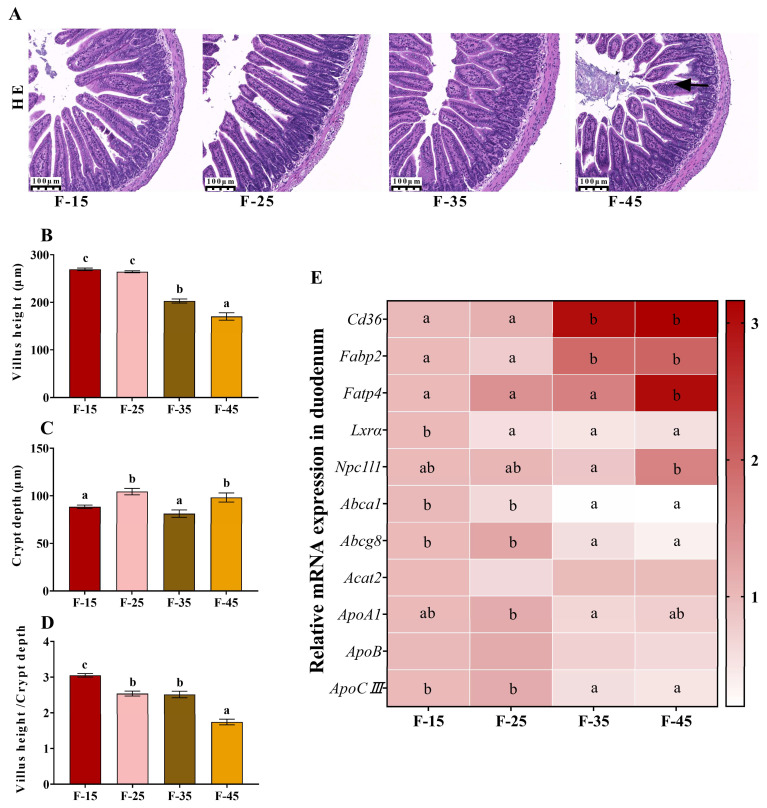
Effect of energy supply from fat on the structure of the ileum and expression of genes regulating lipid absorption in the duodenums in mice. (**A**) Representative images of H&E-stained ileum tissues (magnification 100×, scale bar = 100 μm), n = 3 for each group. (**B**) Villus height. (**C**) Crypt depth. (**D**) Villus height/ Crypt depth. (**E**) The mRNA expression levels of genes associated with lipid absorption and efflux in the duodenums of mice, n = 5 for each group. Data are presented as the mean ± SEM. Different superscript letters (a–c) indicate significant differences.

To evaluate the absorption of fatty acids and cholesterol in the small intestine, tissue sections of the small intestine were examined via histological analysis, and the expressions of mRNA and proteins related to fatty acid absorption and transport in the duodenum were evaluated. In the F-15, F-25, and F-35 groups, the structure of the ileum was normal, mucosal epithelial cells were normal in structure and tightly arranged, and cell degeneration and shedding or the infiltration of inflammatory cells were not observed (Figure 4A). However, the structure of the ileum was slightly abnormal and vacuolation was observed in some epithelial cells in the mucosal layer. The height of villi was significantly higher in the F-15 and F-25 groups than in the F-45 group, and the villus height-to-crypt depth ratio was lowest in the F-45 group (*p* < 0.01) (Figure 4B–D). These results suggest that high dietary fat intake adversely affects intestinal structure. The expressions of *Cd36* and *Fabp2* were higher in the F-35 and F-45 groups than in the F-15 and F-25 groups (*p* < 0.01). The expression of *Fatp4* was highest in the F-45 group (*p* < 0.01). In addition, the expressions of *Abca1* and *Abcg8* genes, which are associated with cholesterol efflux, were reduced in the F-45 group (*p* < 0.05). However, the mRNA expressions of *Lxr* and *ApocⅢ* in the small intestines of mice were significantly higher in the F-15 group than in the F-35 and F-45 groups (*p* < 0.05).

### 2.4. The Effect of Different Fat Intake Levels on the Diversity of Intestinal Microbiota

**Figure 5:**
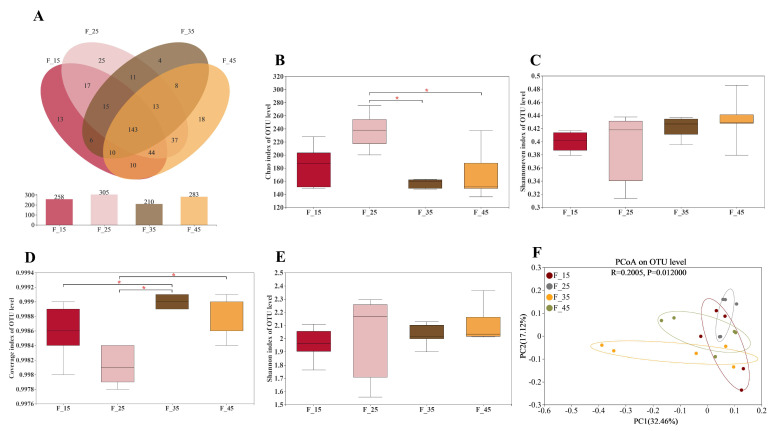
Comparison of α and β diversity among groups. (**A**) Species composition at OTU level. (**B**) Chao index. (**C**) Shannoneven index. (**D**) Coverage index. (**E**) Shannon index. (**F**) The principal coordinates analysis (PCoA). For all groups, n = 5 for each group. * *p* < 0.05.

16S rRNA gene sequencing was performed to examine the influence of blended oils with different energy supply levels on the composition of the gut microbiota. The number of OTUs was 258, 305, 210, and 283 in the F-15, F-25, F-35, and F-45, respectively (Figure 5A). The total number of OTUs in all groups was 143. The highest read amounts in intestinal microbiota OTUs were observed when the energy supply from fat was 25%. Very high or very low levels of dietary energy supplied from fat led to a reduction in the number of OTUs. As shown in Figure 5B, the richness of the intestinal microbiota measured based on OTUs (Chao index) was significantly higher in the F-25 group than in the F-35 and F-45 groups (*p* < 0.05). The Shannon and Shannoneven indices, indicating community diversity and evenness among groups at the OTU level, respectively, were not significantly different among the four groups (*p* > 0.05) (Figure 5C,E). The coverage index was significantly lower in the F-25 group than in the F-35 and F-45 groups at the OTU level (*p* < 0.05) (Figure 5D). The principal coordinate analysis (PCoA) revealed a significant difference in microbiome diversity among the four groups (R^2^ = 0.2005, *p* = 0.012) (Figure 5F). These results suggest that different levels of energy supply from fat lead to differences in the diversity of the intestinal microbiota.

### 2.5. The Effect of Different Fat Intake Levels on the Composition of Intestinal Microbiota

**Figure 6:**
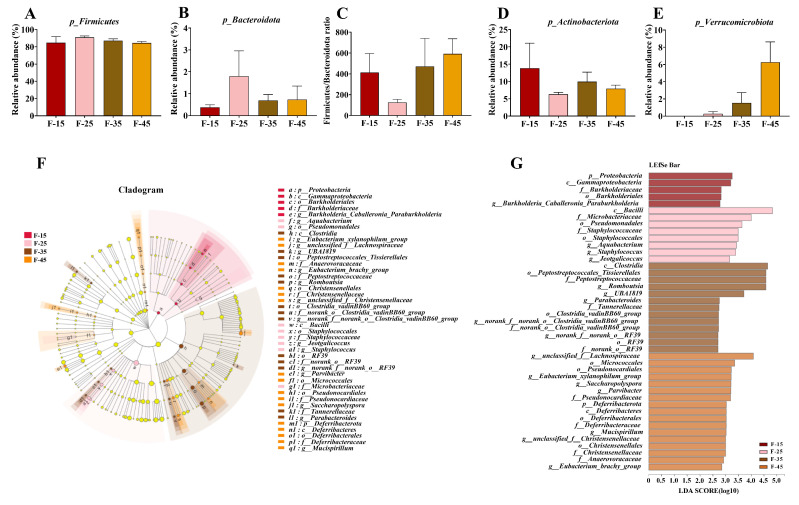
Relative abundance of gut microbes at the phylum and genus levels. (**A**,**B**) Relative abundance of *Firmicutes* and *Bacteroidota* at the phylum level. (**C**) F/B ratio. (**D**,**E**) Relative abundance of *Actinobacteriota* and *Verrucomicrobiota* at the phylum level. (**F**,**G**) Linear discriminant analysis with effect size measurement (LEfSe) for estimating multi-level species differences.

**Figure 7:**
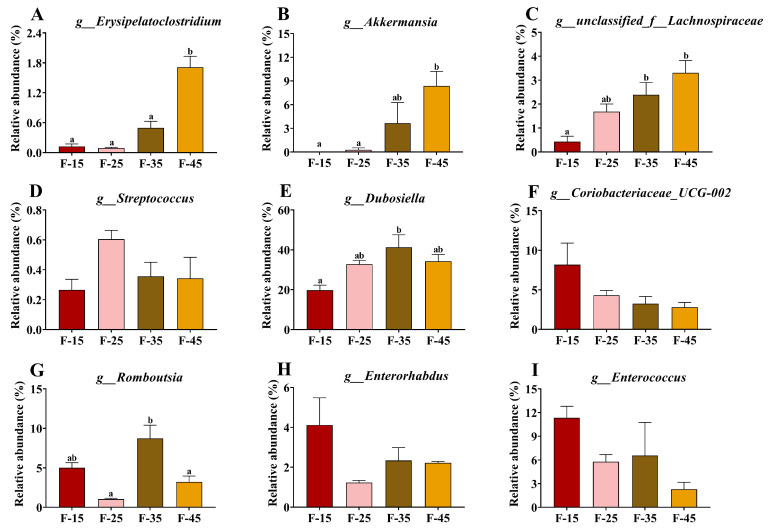
Relative abundance of gut microbes at the genus and genus levels. (**A**–**I**) Relative abundance of *Erysipelatoclostridium*, *Akkermansia*, *unclassified_f__Lachnospiraceae*, *Staphylococcus*, *Dubosiella*, *Coriobacteriaceae_UCG-002*, *Romboutsia*, *Enterorhabdus*, and *Enterococcus* at the genus level. For all groups, n = 5. Data are expressed as the mean ± SEM. Different superscript letters (a and b) indicate significant differences.

**Figure 8:**
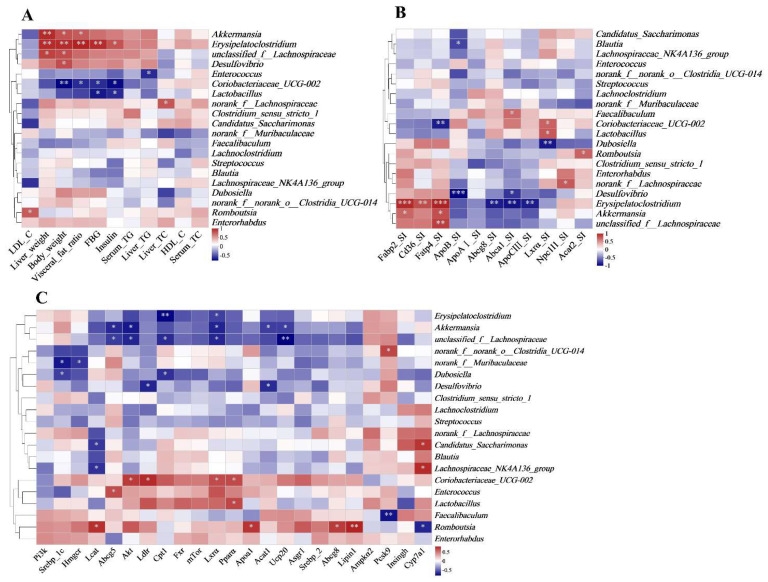
Correlation among phenotypes, genes, and the abundance of key microbial species. (**A**) Heatmap demonstrating the correlation between the abundance of intestinal microbes and glucose levels, final body weight, visceral fat ratio, and final glucose. (**B**) Heatmap demonstrating the correlation between the abundance of intestinal microbes and the expression of genes associated with glucose and lipid absorption and transport in the duodenum. (**C**) Heatmap demonstrating the correlation between the abundance of intestinal microbes and the expression of genes associated with glucose and lipid metabolism in the liver. For all groups, n = 5. * *p* < 0.05; ** *p* < 0.01; *** *p* < 0.001.

Figure 6 demonstrates the effects of different levels of energy supplied from fat on intestinal microbiota at the phylum and genus levels. At the phylum level, *Firmicutes*, *Actinobacteria*, *Verrucomicrobiota*, and *Bacteroidota* were the most abundant bacteria (Figure 6A–G). The abundance of *Proteobacteria* was highest in the F-15 group, whereas *Deferribacterota* was highest in the F-45 group (Figure 6F,G). At the genus level, significant differences were observed in the relative abundance of *Erysipelatoclostridium*, *Akkermansia*, *unclassified_f__Lachnospiraceae, Dubosiella*, and *Romboutsia* among the four groups (Figure 7A–I). The F-15 group had the lowest abundance of *Erysipelatoclostridium*, *Akkermansia, Dubosiella*, and *unclassified_f_Lachnospiraceae* and the highest abundance of *Coriobacteriaceae_UCG-002*, *Romboutsia, Enterorhabdus*, and *Enterococcus*, whereas the F-45 group had the highest abundance of *unclassified_f_Lachnospiraceae*, *Erysipelatoclostridium*, and *Akkermansia* (Figure 7A–I). Furthermore, the relationship between lipid metabolism and gut microbial diversity was assessed through a correlation analysis. The relative abundance of *Erysipelatoclostridium*, *Akkermansia*, and *unclassified_f__Lachnospiraceae* was significantly positively correlated with liver weight, body weight, insulin levels, visceral fat ratio, and FBG. However, the relative abundance of *Staphylococcus, norank_f_Muribaculaceae, norank_f_ norank_o_Clostridia_UCG_014*, and *Dubosiella* was significantly negatively correlated with TC levels of the livers in mice (Figure 8A). The expression of mRNA for cholesterol synthesis-related genes such as *Lxrα*, *Srebp-2*, *mTor*, *Acat1*, and *Ucp20* in the livers of mice was negatively correlated with the relative abundance of *Erysipelatoclostridium*, *Akkermansia*, *Staphylococcus*, *Dubosiella*, and *unclassified_f__Lachnospiraceae*; however, it was significantly positively correlated with the relative abundance of Coriobacteriaceae_UCG-002, *Romboutsia*, *Enterorhabdus*, and *Enterococcus* (Figure 8C). The expression of mRNA for fatty acid transporters such as *Fatp4*, *Fabp2*, and *Cd36* in the small intestines of mice were significantly positively correlated with the relative abundance of *Erysipelatoclostridium*, *Akkermansia*, and *unclassified_f__Lachnospiraceae*; however, it was significantly positively correlated with the relative abundance of Coriobacteriaceae_UCG-002. The relative abundance of *Erysipelatoclostridium* was significantly negatively correlated with the mRNA expressions of *Abcg8*, *Abca1*, and *ApcoⅢ* in the small intestines of mice (Figure 8B).

**Figure 9:**
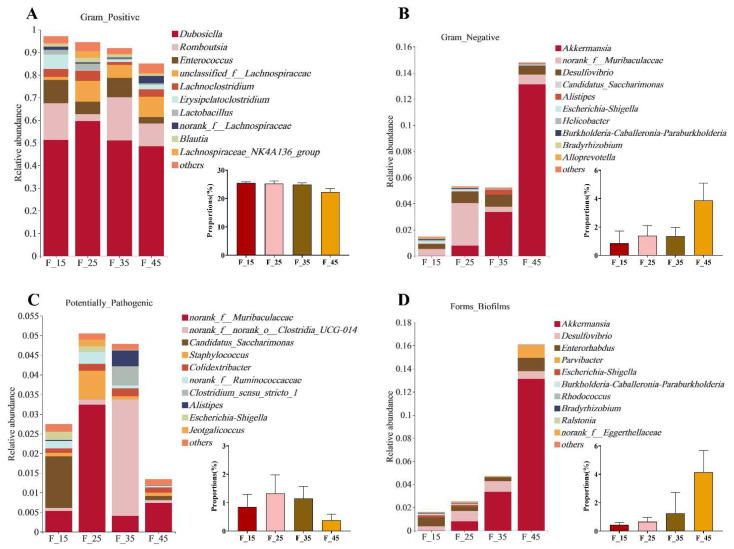
Species contribution and differences in the abundance of Gram-positive, Gram-negative, potentially pathogenic, and biofilm-forming bacteria at the genus. (**A**) Species contribution of Gram-positive bacteria and differences in their abundance. (**B**) Species contribution of Gram-negative bacteria and differences in their abundance. (**C**) Species contribution of potentially pathogenic bacteria and differences in their abundance. (**D**) Species contribution of biofilm-forming bacteria and differences in their abundance. For all groups, n = 5.

The BugBase platform was used to analyze and predict phenotypes in the gut microbiota at the genus level. The predicted microbial phenotypes were divided into seven categories as follows: Gram-positive, Gram-negative, biofilm formation, potentially pathogenic, containing mobile elements, oxygen utilizing, and stress tolerant. At the genus level, the top 9 Gram-positive bacterial species in each group were *Dubosiella, Romboutsia, Enterococcus, unclassified_f_Lachnospiraceae, Lachnoclostridium, Lactobacillus, norank_f__Lachnospiraceae, Blautia*, and *Lachnospiraceae_NK4A136_group* (Figure 9A); and the top 10 Gram-negative bacterial species in each group were *Akkermansia, norank_f__Muribaculaceae, Desulfovibrio, Candidatus_Saccharimonas, Alistipes, Escherichia-Shigella, Helicobacter, Burkholderia-Caballeronia-Paraburkholderia, Bradyrhizobium*, and *Alloprevotella.* No significant differences were observed in the proportion of Gram-negative species among the four groups (Figure 9B). The top 10 potentially pathogenic species in each group were *norank_f__Muribaculaceae, norank_f__norank_o__Clostridia_UCG-014, Candidatus_Saccharimonas, Staphylococcus, Colidextribacter, norank_f__Ruminococcaceae, Clostridium_sensu_stricto_1, Alistipes, Escherichia-Shigella*, and *Jeotgalicoccus* (Figure 9C). The top 10 biofilm-forming species in each group were *Akkermansia, Desulfovibrio, Enterorhabdus, Parvibacter, Escherichia-Shigella, Burkholderia-Caballeronia-Paraburkholderia, Rhodococcus, Bradyrhizobium, Ralstonia*, and *norank_f__Eggerthellaceae* (Figure 9D). No significant differences were observed in the proportion of Gram-positive, Gram-negative, potentially pathogenic, and biofilm-forming species among the four groups. The above results indicate that different dietary patterns can have a greater impact on the composition of intestinal microbiota in mice, and there is a certain link between these changes in intestinal microbiota and liver lipid metabolism in mice.

## 3. Discussion

A high-fat and high-calorie diet is closely associated with the development of metabolic diseases such as obesity, diabetes, and NAFLD [12]. In contrast, the relationship between an HCLF diet and metabolic diseases remains controversial. In this study, we compared the effects of different carbohydrate and fat intake levels on metabolism in mice. Body weight, visceral fat deposition, serum lipid levels, and TG levels in the liver were higher among mice fed a high-fat and high-calorie diet (F-45 group) than among mice fed an HCLF diet (F-15 group). However, TC content in the liver was highest in mice fed an HCLF diet. Yan et al. [11] reported that an HCLF diet led to lipid metabolism disorders and NAFLD in mice after 16 weeks but did not induce obesity. Tessitore A et al. [12] demonstrated that long-term intake of an HCLF diet induced the development of hepatocellular cancer in mouse models of NAFLD/NASH without causing weight gain. Pompili S et al. [10] reported that an HCLF diet was equally as effective as a high-fat diet (HFD) in developing NAFLD in mice. HCLF diets can effectively reduce body weight without calorie restriction [24,25,26]. Hu S et al. [27] reported that fat macronutrients, not proteins or carbohydrates, led to adiposity, which is consistent with the results of this study. However, an HCLF diet induced total cholesterol deposition in the livers of mice, but liver weight and serum levels of ALT were lower in this group of mice than in the high-fat group. We hypothesized that the reason why the liver weights of mice in the HCLF diet group were lower than those in the HFD group was related to the body weights of the mice because our results showed that the liver indices of the two groups of mice were similar. This result was consistent with the findings of Pompili et al. [10]. The enzyme activity of ALT in the HCLF group of mice was lower than that in the HFD group and is probably related to the feeding cycle of the mice. It might be that the feeding cycle of mice was not long enough (only 6 months) in our experiment. Although mice in the HFLD group showed cholesterol deposition, it did not cause serious damage to hepatocytes. Pompili et al. [10] demonstrated that prolonged LF-HCD induced the same effect as an isocaloric HFD in a nutritional mouse model of NAFLD/NASH after feeding mice with an HCLF diet and HFD diet for 18 months.

However, which HCLF diets contribute to the increase in TC content in the livers of mice remains unknown. To investigate this underlying mechanism, we examined the mRNA expression of genes related to fatty acid and cholesterol metabolism in the liver and genes related to lipid absorption and transport in the small intestines of mice. Luo et al. [28] indicated that cholesterol metabolism includes four main parts: endogenous synthesis, exogenous uptake, efflux, and esterification. First, cholesterol is mainly synthesized in the liver, with 3-hydroxy-3-methylhydroxypentanedioyl-CoA reductase (HMGCR) being the rate-limiting enzyme of the endogenous synthesis pathway, and increased mRNA expression of *Hmgrc* causes increased cholesterol synthesis in the liver [29]. *Srebp-2* is a key factor for cholesterol synthesis, and up-regulation of *Srebp-2* stimulates the gene expression of both *Ldlr* and *Hmgrc* [30]. Serum LDL-C is internalized by hepatocytes through *Ldlr*, so up-regulation of the expression of the *Ldlr* gene may promote the absorption of serum cholesterol by hepatocytes [31]. In addition, Lu et al. [32] reported that feeding induces cholesterol biosynthesis via the mTORC1-USP20-HMGCR axis. Wang et al. [33] similarly showed that activation of ASGR1 upregulates *mTorc1* expression. Lipin-1 is a key metabolic enzyme of the fat synthesis pathway [34], and mTORC1 can upregulate SREBP2 protein expression by phosphorylating lipin1 and preventing its nuclear entry [28]. Cholesterol 7α-hydroxylase (CYP7A1) is the rate-limiting enzyme in cholesterol metabolism, and cholesterol is transferred to high-density lipoprotein (HDL) particles and returned to the liver for conversion into bile acids (Bas) predominantly via the CYP7A1 [35]. In this study, the mRNA expression of *Asgr1*, *mTorc1*, *Ucp20*, *Srebp2*, *Hmgcr*, and *Ldlr* was higher in mice on an HCLF diet (F-15 group) than in mice on a high-fat and high-calorie diet (F-45 group). The intake of HCLF-diet-induced hepatic cholesterol synthesis in mice through the ASGR1-mTORC1-USP20-HMGCR signaling pathway may be one of the reasons for the highest cholesterol content in the livers of mice in the F-15 group. Moreover, in this study, Acat1 expression in the liver was higher in the F-15 group. Acetyl coenzyme acetyltransferase 1 (Acat1) is a key enzyme catalyzing lipid synthesis in the endoplasmic reticulum in the liver. Its primary function in the liver is to convert free cholesterol into cholesteryl esters, and its elevated expression can increase the content of total and esterified cholesterol in the liver [36]. This phenomenon may explain why the cholesterol content in the liver in the F-15 group was highest. Increased cholesterol levels in hepatocytes stimulate *Lxrα* expression, and up-regulation of *Lxrα* stimulates ABCG5/ABCG8 transporter activation, thereby enhancing cholesterol efflux into bile [37]. Our study yielded consistent results, such as the upregulation of *Abcg8* mRNA expression. However, *Npc1l1* expression did not significantly differ among the four groups. In our study, it is meaningful to find that the mRNA expression of *Lxrα* in the liver was significantly higher in the HCLF group than the other three groups because *Lxrα* is a sterol sensor that can regulate intracellular cholesterol levels and induce the expression and activation of *Srebp-1c* [38]. *Srebp-1c* is positively regulated through the transcription factor *Lxrα*, which forms a heterodimer with retinoid X receptor α [39]. In addition, stimulation of peroxisome proliferator-activated receptor alpha (*Pparα*) increases the mRNA expressions of *Lxrα* [30,39,40], the accumulated fatty acids in the liver, which are endogenous *Pparα* agonists, may increase *Lxrα* expression [41]. Accumulation of fatty acids in the liver upregulates the expression of *Pparα*, whereas activation of *Pparα* activates *Lxrα*. Additionally, upregulation of *Lxrα* promotes the upregulation of *Srebp-1c*, thereby promoting the synthesis of fatty acids; upregulates the expression of *Cpt1* (the rate-limiting enzyme of fatty acid β-oxidation); and promotes the oxidation of fatty acids, which can explain the lower TG content in the liver in the F-15 group than in the F-45 group. *Tnf-α* is not only a marker of inflammation but also a known risk factor for the development of NAFLD [42]. *Il-1β* and *Il-6* are two important pro-inflammatory cytokines that amplify inflammation and sensitize hepatocytes to TNF-induced liver damage [43]. ADAMTS1, a metalloproteinase belonging to the ADAMS family, is involved in extracellular matrix (ECM) remodeling [44]. Several studies suggest a link between ADAMTS1 not only with the development of atherosclerotic plaques and ATH [45] but also with the ability to activate hepatic fibrotic TGF-b [46]. *Col4a1* is a hepatic fibrosis-associated gene, and the expression of its mRNA induces hepatic fibrosis, which can result in liver injury [47]. Our study showed that the mRNA expression levels of *Col4a1*, *Adamts1*, and *Il-1β* in the livers of mice in the HCLF group were significantly higher than those in the HFD group. This result suggests that the intake of the HCLF diet increases the risk of liver inflammation and fibrosis, but is not consistent with the above results of ALT activity in the serum.

The intestine and liver are anatomically connected by the hepatic portal system, also known as the gut-liver axis. The gut microbiota and their metabolic products may influence liver pathology [48,49]. Diet plays an important role in shaping the composition and function of the intestinal microbiota in humans and rodents [37,50]. Therefore, we analyzed the effects of diets with different ratios of carbohydrates and fats on the microbial diversity of the small intestine, the associated phenotypes, and the mRNA expression of genes. Alpha diversity reflects the species richness of the microbial community and can be evaluated based on several indices, including the Chao, Shannon, Shannoneven, and coverage indices [51,52]. Increasing studies have shown that the reduced *Firmicutes/Bacteroidetes (F/B) ratio* was associated with NASH and most of that was significantly associated [53] and *Actinobacteriota* were significantly correlated with liver steatosis [54]. In our results, compared with the level of 25% fat energy, HCLF diets consistent with HCHF decreased the F/B ratio. However, the abundance of *Actinobacteriota* was increased compared with the other three groups. Rodrigues et al. [55] reported the presence of *Romboutsia* species in the intestines of >80% of patients with obesity. The *Romboutsia* genus may be a ubiquitous microbial community in people who are overweight, and reducing its abundance can play a role in alleviating obesity [56]. In our results, HCLF diets consistent with HCHF significantly increased the abundance of *Romboutsia.* Moreover, it has been shown that *Akkermansia* has the ability to produce SCFAs [57], which are effective molecules to lower cholesterol levels [58], and that they reduce cholesterol levels in the host through five major pathways [59]. First, SCFAs can reduce plasma cholesterol levels in mice by downregulating the expression of *Srebp2* and *Hmgcr*, thereby reducing the rate of cholesterol synthesis in the liver and small intestine [60,61]. Second, up-regulation of Cyp7a1 by SCFAs promotes the conversion of cholesterol to Bas, thereby reducing cholesterol levels [58]. Third, SCFAs could inhibit intestinal cholesterol absorption by decreasing the expression of Npc1l1 and increasing the expression of Abcg5/8 in mouse intestinal tissues [62]. Fourth, SCFAs can accelerate cholesterol export from the liver by regulating ATP-binding cassette transporter A1 (ABCA1) [62]. Fifth, SCFAs can also affect cholesterol levels through signaling pathways mediated by G protein-coupled receptors 41 and 43 (GPR41/43) [63]. In addition, Wang et al. [64] reported that the abundance of *unclassified_f_Lachnospiraceae* was significantly reduced in the mice models of methotrexate-induced hepatoxicity. However, Li et al. [65] reported that the abundance of *unclassified_f_Lachnospiraceae* was significantly high in mice models of high-fat diet-induced glucose metabolism disorders. Gallates have been used to reduce the abundance of *unclassified_f_Lachnospiraceae* and improve immune function in the ileum of mice fed an HFD [66]. In this study, the abundance of *Akkermansia* and *unclassified_f_Lachnospiraceae* in the intestines of mice in the F-15 group was significantly lower than that in the F-45 group, and they were significantly positively correlated with body weight, liver weight, and *Fabp2* and significantly negatively correlated with the mRNA expression of *Lxrα, Srebp-2, mTor, Acat1*, and *Ucp20* in the livers of mice. It may be the reason why hepatic cholesterol content was higher in mice fed the HCLF diet than in the high-fat group. The results of this study showed that the HCLF diet induced hepatic cholesterol deposition more easily in mice than a high-fat diet. It has been shown that the HCLF diet can be used in studies of lean NAFLD. Moreover, the HCLF diet is better suited to mimic Asian dietary conditions to interrogate NAFLD pathogenesis than a high-fat diet. The reason for this lies in the different dietary conditions, with high-fat diets predominating in the United States and Europe, while high-carbohydrate diets predominate in Asia [11].

## 4. Methods and Materials

### 4.1. Experimental Design and Feed Formulation

Eight-week-old male C57BL/6J mice (n = 60) were purchased from Hunan SJA Laboratory Animal Co., Ltd. (Changsha, China) (SCXK (Xiang) 2019-0004). All experimental animals in a Special Pathogen Free (SPF) environment were fed on a regular diet for one week and randomly divided into four groups (n = 15 per group) according to body weight. The animals in the different groups were fed on purified diets containing 15% (F-15, 62.7% carbohydrate supply, 15% fat supply), 25% (F-25, 52.7% carbohydrate supply, 25% fat supply), 35% (F-35, 42.7% carbohydrate supply, 35% fat supply), and 45% (F-45, 32.7% carbohydrate supply, 45% fat supply) of fat energy for 24 weeks. The purified diets were produced by Trophic Animal Feed High-Tech Co., Ltd. (Nantong, China), according to the American Dietetic Association AIN93M. The diet formulas are shown in Table S1. The mice were housed at a humidity of 50–60%, a temperature of 24–26 °C, and a 12 h light/12h dark cycle at the Hunan Agricultural University. They were allowed ad libitum access to water and diet throughout the experiment.

An oral glucose tolerance test (OGTT) [67] was performed at 22 w. Mice were made to fast without water for 6 h, n = 15, and 3 g/kg oral glucose was given. The blood glucose values of mice were recorded at 0 min, 30 min, 60 min, 90 min, and 120 min after gavage. The area under the curve from the obtained data was calculated to evaluate the glucose tolerance of mice in each group [68]. For the insulin tolerance test (ITT) [69] at 23 w, mice were made to fast without water for 4 h, n = 15. Then, blood glucose was measured, and mice were intraperitoneally injected with insulin at a dose of 0.5 U/kg. Blood glucose levels of each mouse were recorded at 0 min, 30 min, 60 min, 90 min, and 120 min after injection. The area under the curve was calculated to evaluate the insulin resistance of each group of mice. Growth changes were recorded throughout the experiment. All mice were made to fast without water for 6 h and euthanized at the end of 24 weeks. The fasting blood glucose levels, visceral fat rate, serum lipids, liver enzymes, and intestinal microbiota were determined. The liver, duodenum, and ileal were frozen in liquid nitrogen and stored at −80 °C for subsequent experiments. Serum was obtained by centrifuging blood samples at 3 × 10^3^ rpm for 10 min. Samples were then stored at −80 °C for further analysis. All experimental procedures were approved by the Animal Welfare and Use Guidelines of China and the Animal Welfare Committee of Hunan Agricultural University, approval code (43321820).

Food efficiency ratio = feed intake (g)/body weight gain (g) ∗ 100%

Visceral fat ratio = (perirenal fat + epididymal fat) (g)/body weight (g) ∗ 100%

Liver index = weight of the liver (gallbladder removed) (g)/body weight (g)

### 4.2. Determination of Fatty Acid Content in Dietary Oil or Fat

We referred to the national standard of the People’s Republic of China GB5413.27-2010 [70], the food safety national standard for determining fatty acids in infant food and dairy products. Fatty acids in the dietary oil/fat were determined using a gas chromatograph (7890A, Agilent, Santa Clara, CA, USA), Chromatographic column: CD-2560 (100 m × 0.25 mm × 0.20 μm). The method parameters included heating at 130 °C for 5 min, 4 °C/min to 240 °C for 30 min; Injector temperature: 250 °C; Carrier gas flow rate: 0.5 mL /min; Split injection, split: 10:1; Detector: FID; Detector temperature: 250 °C. The results are shown in Table S2.

### 4.3. Biochemical Analysis

Serum triglycerides (TG), total cholesterol (TC), high-density lipoprotein (HDL-C), low-density lipoprotein (LDL-C), alanine aminotransferase (ALT) and aspartate aminotransferase (AST) levels were directly measured using a DS-161V automatic blood biochemical analyzer (SINNOWA Medical Science & Technology, Nanjing, China). Blood glucose levels were determined using a Roche glucometer (ACCU-CHEK, Roche, Shanghai, China). The liver TC and TG levels were determined using commercial kits purchased from Nanjing Jiancheng Bioengineering Institute Co., LTD (Nanjing, China) [68,71].

### 4.4. Histological Analysis

The liver and ileum tissues were collected upon dissecting the mice and were dehydrated and embedded in paraffin. Subsequently, the hepatic and intestinal tissues were subjected to observation of pathological changes through Hematoxylin-eosin staining (H&E-stained), oil-red O staining, and Sirius red staining. Images were obtained using an optical microscope (Nikon Eclipse E100, Nikon, Tokyo, Japan) at 100×/200× magnifications [67,72]. Tissue sections were observed and analyzed using Caseviewer 2.4.0.119028.

### 4.5. RT qPCR

Total RNA was extracted from the tissues using a TRIzol reagent (Thermo Fisher Scientific, Waltham, MA, USA). The RNA was reverse transcribed to complementary DNA (cDNA) using a first-strand cDNA Synthesis Kit (Accurate Biology, Changsha, China). Amplification of RNA from the duodenum was performed using an SYBR Green Tap HS Mixture (Accurate Biology, Changsha, China) in the Real-time qPCR System (Gentier, Tianlong, Xian, China) at the following thermal cycler conditions: 1 cycle at 95 °C for 30 s and 40 cycles at 95 °C for 5 s and at 60 °C for 30 s. However, amplification of RNA from liver samples was performed using a vGreen^®^ Premix Ex Taq^TM^ (Tli RNaseH Plus) (TaKaRa, Beijing, China) in the RT-qPCR System (HiSeq2500, WaferGen Biosystems, Fremont, CA, USA) at the following thermal cycler conditions: 1 cycle at 95 °C for 10 min and 40 cycles at 95 °C for 30 s and at 60 °C for 30 s. Expression of glucose and lipid metabolism-related genes in the duodenum and liver tissues was also determined. β-actin was used for normalization of the target gene expression [71,72]. Sequences of all primers used are shown in Table S3. Relative amounts of mRNA were expressed as the 2^−ΔΔCt^ method.

### 4.6. Western Blotting Analysis

The protein expression levels of ACAT1, UCP20, and SREBP2 were detected by western blotting in the four groups. Western blot analysis was performed as described previously [73]. Equal amounts of protein (50 μg) from each liver sample were electrophoresed on 10% SDS polyacrylamide gels with prestained protein markers. The results were quantitatively analyzed using Image J software (https://imagej.net/ij/) (National Institutes of Health, Bethesda, MD, USA).

### 4.7. Intestinal Microbiota Analysis

Five mice were randomly selected from each group for intestinal microbiota analysis [69]. Microbial community genomic DNA was extracted from the small intestine samples using the FastDNA^®^ Spin Kit for Soil (MP Biomedicals, Irvine, CA, USA) according to the manufacturer’s instructions. The extracted DNA was visualized using 1% agarose gel. The DNA concentration and purity were determined using a NanoDrop 2000 UV-vis spectrophotometer (Thermo Scientific, Waltham, MA, USA). The hypervariable region V3-V4 of the bacterial 16S rRNA gene was amplified using primers 338F (5′-ACTCCTACGGGAGGCAGCAG-3′) and 806R(5′-GGACTACHVGGGTWTCTAAT-3′) in an ABI GeneAmp^®^ 9700 PCR thermocycler (ABI, Foster, CA, USA). Genes were excised from the agarose gel and purified using an AxyPrep DNA Gel Extraction Kit (Axygen Biosciences, Union City, CA, USA) according to the manufacturer’s instructions and quantified using a Quantus™ Fluorometer (Promega, Madison, WI, USA). Purified amplicons were pooled in equimolar amounts and paired-end sequenced on an Illumina MiSeq PE300 platform/NovaSeq PE250 platform (Illumina, San Diego, CA, USA) according to the standard protocols. The raw 16S rRNA gene sequencing reads were demultiplexed and quality-filtered by fast version 0.20.0, and paired reads were merged using FLASH version 1.2.7. Operational taxonomic units (OTUs) with 97% similarity cutoff were clustered using UPARSE version 7.1. Chimeric sequences were identified and removed. The taxonomy of each OTU representative sequence was analyzed by RDP Classifier version 2.2 against the 16S rRNA database (Such as Silva v138) using a confidence threshold of 0.7.

### 4.8. Statistical Analysis

SPSS version 25.0, was used to statistically analyze the experimental data of each group, and all the data were in line with normal distribution. The data were analyzed by one-way ANOVA. The LSD method was used when the variances were uniform, and Tamhanes’ T2 method was used when the variances were uneven. The results were expressed as the mean and standard error of the mean (Mean ± SEM). Duncan’s marking method was used to mark the significance of the groups. There was no significant difference in any group having the same letter (*p* > 0.05), while there was a significant difference in the groups not having the same letter (*p* < 0.05). GraphPad Prism 6.01 software was used for mapping.

Sequencing data were analyzed by the Meiji Biocloud platform, and Alpha diversity and dilution curves used Mothur 1.30.1 analysis, Beta diversity analysis using Qiime 1.9.1 and R language, and community heat map between groups. The different test was calculated by the Wilcoxon sign rank test, and the correlation heat map of environmental factors was calculated by Spearman Rank correlation coefficient and R language analysis.

## 5. Conclusions

High-fat and high-calorie diets (dietary energy ≥ 35% from fat) can cause obesity and accumulation of serum and hepatic lipids in mice, both of which are closely related to the change of gut microbiota. A high-carbohydrate and low-fat diet (62.7% carbohydrate supply, 15% fat supply) does not cause an increase in body weight and body fat percentage in mice but induces liver cholesterol deposition. However, the mechanisms underlying the effects of a high-carbohydrate, low-fat diet are different from those underlying the effects of a high-fat and high-calorie diet. In addition, a high-carbohydrate and low-fat diet can be used as one of the dietary conditions for studying NAFLD, and it is preferable to a high-fat diet when studying lean NAFLD or modeling Asian dietary habits.

## Data Availability

The data that support the findings of this study are available from the corresponding author upon reasonable request.

## Supplementary Materials

The supporting information can be downloaded at: https://www.mdpi.com/article/10.3390/ijms241914700/s1.
