# Supplementary material for: High-Carbohydrate Diet Consumption Poses a More Severe Liver Cholesterol Deposition than a High-Fat and High-Calorie Diet in Mice
Source: Int J Mol Sci. 2023 Sep 28;24(19):14700. doi: 10.3390/ijms241914700 (PMC10572265; doi:10.3390/ijms241914700)
Supplement: Supplementary file 1 [file ijms-24-14700-s001.zip › ijms-2559156-supplementary.pdf]

Supplementary Materials:

Supplementary Table S1. Dietary formulas.

	F-15	F-25	F-35	F-45
Casein, L-Cystine	237.20	251.20	267.00	284.90
Dextrin	316.60	281.90	242.80	198.60
Sucrose	283.00	249.00	210.70	167.30
Soybean oil	31.90	56.40	83.80	115.00
Lard	31.90	56.40	83.80	115.00
Cellulose	50.90	53.90	57.30	61.20
Vitamins, minerals, choline	48.40	51.20	54.40	58.10
TBHQ	0.01	0.02	0.03	0.05
Total	1000	1000	1000	1000
Ingredient gm (kcal/g)	3.8	4.1	4.3	4.6
Protein (kcal %)	22.2%	22.2%	22.2%	22.2%
Carbohydrate (kcal %)	62.7%	52.7%	42.7%	32.7%
Fat (kcal %)	15.0%	25.0%	35.0%	45.0%
Total	100%	100%	100%	100%

Note: 1 kg diet supplemented with 3.1 g alanine, 4.5 g arginine and 25.5 g glutamate, 2.3 g glycine, 9.2 g lysine, 3.3 g methionine, 2.4 g cystine, 1.6 g tryptophan, 14.3 g proline, 6.7 g serine, 3.3 g histidine, 10.9 g leucine, 5.9 g isoleucine, 6.6 g tyrosine, 6.2 g phenylalanine, 4.7 g threonine, 7 g valine, 8 g Aspartic acid, 5000 ppm calcium, 3000 ppm phosphorus, 1033 ppm sodium, 511 ppm magnesium, 45 ppm iron, 35 ppm zinc, 10 ppm fierce, 6 ppm copper, 0.2 ppm iodine, 1 ppm chromium, 300 ppm Inorganic sulfur, 1613 ppm chlorine, 4 IU Vitamin A, 1 IU Vitamin D, 0.86 ppm Vitamin K, 5 ppm Thiamine, B1, 6 ppm riboflavin, 30 ppm niacin, 15 ppm Pantothenic acid, 6 ppm Vitamin B6, 1000 ppm choline, 2 ppm Folic acid, 0.2 ppm biotin and 25ppb Vitamin B1.

Supplementary Table S2. Fatty acid composition of the oils.

**Composition**	**Soybean oil**	**Lard oil**	**Blend oil**
C12:0，g/100g	0.0067	0.1655	0.0861
C14:0，g/100g	0.0963	1.325	0.71065
C16:0，g/100g	11.2237	23.3905	17.3071
C17:0，g/100g	0.0514	（<0.000325）	0.0257
C18:0，g/100g	4.5769	12.8156	8.69625
C20:0，g/100g	0.4093	（<0.000408）	0.20465
C22:0，g/100g	0.4974	（<0.000481）	0.2487
C16:1，g/100g	0.0764	1.631	0.8537
C18:1n9c，g/100g	23.4902	39.1915	31.34085
C20:1，g/100g	4.7844	（<0.000408）	2.3922
C22:1n9，g/100g	0.101	（<0.000500)	0.0505
C18:2n6c，g/100g	49.471	16.7741	33.12255
C20:2，g/100g	（<0.000590）	0.282	0.141
C18:3n6，g/100g	0.432	（<0.000433）	0.216
EPA C20:5n3，g/100g	0.4454	（<0.000766）	0.2227

Supplementary Table S3. Primer sequences.

Gene	Primer sequences (5'-3')	Primer length (bp)	Gene ID
β-actin	F: CATCCGTAAAGACCTCTATGCCAAC<br>R: ATGGAGCCACCGATCCACA	171	NM_007393.5
Srebp-1c	F: TGCGGCTGTTGTCTACCATA<br>R: CCAGGTTGAGGGCACTTAGT	104	NM_001358315
Pi3k	F: CCACGGTTTGGACTATGGAAG<br>R: AGTAGTGGGCTTGGGTGGTT	131	NM_001024955.2
Akt	F: ATAACGGACTTCGGGCTGTG<br>R: TCGAACAGCTTCTCGTGGTC	209	NM_001165894.2
Insig1	F: TTAGCAGCCCTCTCTTTGGG<br>R: ATCAGGGGACGTGTACTGGTAG	144	NM_153526.5
Srebp2	F: ACCTCACGGGGGACTCTG<br>R: CTGGCTGAATGACCGCTGTA	293	NM_033218.2
Asgr1	F: TTGCTGGTGGTTGTCTGTGT<br>R: ACTACTTCCCTGGGTGCTCA	138	NM_009714.3
Lipin1	F: TGCTCGTGAATCCTCTTGGTTC<br>R: CGGGGTTCAGTCCCTTGTAG	100	NM_172950.3
Ucp20	F: GTTGCGACGGCAGGTACT<br>R: GTGGGCATTCTCTTGGGACA	80	NM_028846.5
Pparα	F: TGGTGTTCGCAGCTGTTTTG<br>R: AGATACGCCCAAATGCACCA	153	XM_030248422.2
Cpt-1	F: AGCCAGACGAAGAACATC<br>R: CCTTGACCATAGCCATCC	172	XM_036161417.1
mTorc1	F: CGCTACTGTGTCTTGGCATC<br>R: GTTCATGCTGCTTAGTCGGC	153	NM_020009.2
Ampk	F: ACGATGAGGCTGTGAAGGAA<br>R: CTGATGGAGGACTAGAGGCG	180	NM_001356568.1
Lxrα	F: TCAAGGGAGCACGCTATGTCT<br>R: CCTCTTCTTGCCGCTTCAGT	174	XM_006499168.4

Gene	Primer sequences (5’-3’)	Primer length (bp)	Gene ID
Fxr	F: GCTTGATGTGCTACAAAAGCTG<br>R: CGTGGTGATGGTTGAATGTCC	110	NM_001385711.1
Hmgcr	F: CCCGTCGTGACCTCAAAGAA<br>R: CAGAAGCCCCAAGCACAAAC	193	NM_001360165.1
Cyp7a1	F: GGGATTGCTGTGGTAGTGAGC<br>R: GGTATGGAATCAACCCGTTGTC	100	NM_007824.3
Acat1	F: CGGCTGTCAAAATCTGGGGA<br>R: AGTGGCACCCAACTTCCTTC	195	NM_009230.3
Ldlr	F: AGAAGACTCATGCAGCAGGAAC<br>R: TGCATGTCTCTGGGGACTCA	122	NM_001252659.1
Psck9	F: AACAGCTACAGCTACAGCCC<br>R: CGGGAAGGACATCATCGGAG	38	XM_006498747.2
Apob	F: TGGCTCTGATCCCAAATCCCT<br>R: CCGTGCATTCATTGTCGATCTG	195	NM_009693.2
Abcg8	F: CTGTGGAATGGGACTGTACTTC<br>R: TGTTGTCACTTTCCGAGGAGA	76	NM_026180.3
Abcg5	F: CCTGCTGAGGCGAGTAACAA<br>R: TGGCACCCACAAGCTGATAG	179	NM_031884.2
Lact	F: CCGTGGCTGCACTCTATGAA<br>R: CATGGGCAGCAAATGTACGG	110	NM_008490.2
Fabp2	F: GCATGTGAGGCGGTTAGGTT<br>R: GAGGTCCAGTCCTGTCCACT	113	NM_007980.3
Cd36	F: AGATGACGTGGCAAAGAACAG<br>R: CCTTGGCTAGATAACGAACTCTG	188	NM_001159558.1
Fatp4	F: TGCTCCTGTACTTGGGGTCT<br>R: AATCAGGGCTGTCTTGTCCG	194	NM_011989.5
Npc1l1	F: TGGACATACGCTCAGGCATC<br>R: ATGTGGGACACGAACTCCAC	157	NM_207242.2
Abca1	F: GCCGTCTTTCCAGGACAGTATG<br>R: CAGGGTGGCTCTTCTCATCAAT	100	NM_013454.3

Gene	Primer sequences (5'-3')	Primer length (bp)	Gene ID
Acat2	F: GGGATTCGGCTTCTGTTACGA<br>R: CAGCTGGAATACCTACCCCAC	172	NM_009338.3
Apoa1	F: TGACTCGGGACTTCTGGGAT<br>R: GGAATTCGTCCAGGTAGGGC	114	NM_009692.4
ApoCIII	F: GTCCCCTGTCTTTTCCTGTCT<br>R: TCCCTCTACCTCTTCAGCTCG	125	NM_001289756.1
Il-1β	F: GCAACTGTTCCTGAACTCAACT<br>R: ATCTTTTGGGGTCCGTCAACT	89	NM_008361.4
Il-6	F: TGATGGATGCTACCAAACTGGA<br>R: TGTGACTCCAGCTTATCTCTTGG	197	NM_001314054.1
Tnfα	F: CCCTCACACTCAGATCATCTTCT<br>R: GCTACGACGTGGGCTACAG	61	NM_013693.3
Col4a1	F: CACCCATCTCTGGGGACAAC<br>R: GTTAGGGCACTGCGGAATCT	116	NM_009931.2
Adamts1	F: GGGGAGACTGCTCAAGAACC<br>R: ACCTGTAGCGGACTCGTTTG	117	NM_009621.5

Supplementary Method S1. Liver total cholesterol and triglyceride content analysis.

1). Sample preparation: 0.1 g liver tissues were weighed accurately with an analytical balance, add 9 times volume of normal saline, ground at 60 Hz for 1 min at 4℃ to prepare 10% homogenate, centrifuged at 3000 r/min and 4℃ for 10 min, and the supernatant was aspirated for testing.

2) Determination of protein content. The concentration of total protein in each set of samples was tested according to the BCA kit instructions. The procedure is as follows.

	**Blank well**	**Standard well**	**Assay well**
Double distilled water（μL）	10	-	-
563 μg/mLStandard（μL）	-	10	-
Samples to be tested（μL）	-	-	10
Working solution（μL）	250	250	250
Mix well, incubate at 37℃ for 30 minutes at 562 nm, and measure the absorbance of each well by microplate reader.			

Microplate reader colorimetric calculation formula: Total protein concentration (μg/mL) = (measured OD value - blank OD value) / (standard OD value - blank OD value) * calibrator concentration (524 μg/mL) * dilution factor before sample test

3) Determination of total cholesterol. According to the TC kit instructions, the content of cholesterol in mouse liver tissue was detected. The operation steps are as follows:

	**Blank well**	**Standard well**	**Assay well**
Double distilled water（μL）	2.5	-	-
Standard（μL）	-	2.5	-
Samples to be tested（μL）	-	-	2.5
Working solution（μL）	250	250	250
Mix well, incubate at 37℃ for 10 minutes at 510 nm, and measure the absorbance of each well by microplate reader.			

Microplate reader colorimetric calculation formula: Total cholesterol content (mmol/gprot) = (sample OD value - blank OD value) / (standard OD value - blank OD value) * calibrator

concentration (mmol/L) / protein concentration of sample to be tested (gprot/L)

4) Triglyceride determination. According to the instructions of TG kit, the content of triglyceride in liver tissue of mice was detected. The operation steps are as follows:

	**Blank well**	**Standard well**	**Assay well**
Double distilled water（μL）	2.5	-	-
Standard（μL）	-	2.5	-
Samples to be tested（μL）	-	-	2.5
Working solution（μL）	250	250	250
Mix well, incubate at 37℃ for 10 minutes at 510 nm, and measure the absorbance of each well by microplate reader.			

Microplate reader colorimetric calculation formula: Triglyceride content (mmol/gprot) = (sample OD value - blank OD value) / (standard OD value - blank OD value) * calibrator concentration (mmol/L) / protein concentration of the sample to be tested (gprot/L)

5) Accuracy:

a. the absorbance of the reagent blank tube was ≤ 0.100.

b. linearity: r 2 > 0.995 in the range of 0 to 19.39 mmol/L.

c. precision: CV ≤ 3% and inter-assay relative range ≤ 5%.

6) Each set of 15 replicates was tested three times, and the trends of the analysis results were consistent for the three times.
